# *In silico* integrative analysis of multi-omics reveals regulatory layers for diurnal gene expression in mouse liver

**DOI:** 10.3389/fendo.2022.955070

**Published:** 2022-07-22

**Authors:** Chunjie Jiang, Panpan Liu, Cam Mong La, Dongyin Guan

**Affiliations:** Division of Diabetes, Endocrinology, and Metabolism, Department of Medicine, Baylor College of Medicine, Houston, TX, United States

**Keywords:** diurnal rhythm, multi-omics analysis, liver, gene expression, regulatory layer

## Abstract

Diurnal oscillation persists throughout the body and plays an essential role in maintaining physiological homeostasis. Disruption of diurnal rhythm contributes to many diseases including type 2 diabetes. The regulatory mechanism of the transcription-translation feedback loop (TTFL) of core clock genes is well-established, while a systematic study across all regulatory layers of gene expression, including gene transcription, RNA translation, and DNA binding protein (DBP) activities, is still lacking. We comprehensively bioinformatics analyzed the rhythmicity of gene transcription, mature RNA abundance, protein abundance and DBP activity using publicly available omic-datasets from mouse livers. We found that the core clock genes, *Bmal1* and *Rev-erbα*, persistently retained rhythmicity in all stages, which supported the essential rhythmic function along with the TTFL. Interestingly, there were many layer-specific rhythmic genes playing layer-specific rhythmic functions. The systematic analysis of gene transcription rate, RNA translation efficiency, and post-translation modification of DBP were incorporated to determine the potential mechanisms for layer-specific rhythmic genes. We observed the gene with rhythmic expression in both mature RNA and protein layers were largely due to relatively consistent translation rate. In addition, rhythmic translation rate induced the rhythms of protein whose mature RNA levels were not rhythmic. Further analysis revealed a phosphorylation-mediated and an enhancer RNA-mediated cycling regulation between the corresponding layers. This study presents a global view of the oscillating genes in multiple layers *via* a systematical analysis and indicates the complexity of regulatory mechanisms across different layers for further functional study.

## Introduction

Diurnal rhythms are approximate 24-hour cycles in which there are regular light and dark periods (1). These rhythms are critical to maintaining physiological homeostasis *via* aligning the internal clock with daily environmental changes, such as light and temperature (2, 3). The disruption of diurnal rhythms due to shift work or sedentary lifestyle leads to many diseases, including metabolic disorders and various cancers (4–6) and is associated with the development of type 2 diabetes, a global health problem (7, 8). Understanding the regulatory mechanism underlying diurnal rhythms could shed light on potential chrono-therapeutic strategies and druggable targets.

Several transcription activators and repressors form the transcription-translation feedback loop (TTFL). The transcription activators include brain and muscle ARNT-like 1 (BAML1) and circadian locomotor output cycles kaput (CLOCK), and transcription repressors include REV-ERB (9–12). Due to the central role of TTFL in the expression of core clock genes, TTFL is considered to be the universal building block of circadian clocks (13). In addition to core clock genes, the question of how oscillating genes are regulated is still largely unknown.

With advanced technologies of next-generation sequencing and mass spectrometry, emerging studies established genome-wide datasets regarding oscillating enhancer activity (4, 14), gene transcription (14), mature RNA abundance (15), protein level (16–18), and DNA binding protein (DBP) activity (18). Moreover, the rhythms of mature RNA translation rate (19) and phosphorylation (20) have been recently measured. These datasets provide us an opportunity to systematically determine the conservativity and specificity of diurnal rhythm for gene expression in various layers, including RNA transcription, processing translation, and protein post-translation modification and activities. The pathways enriched in layer-specific rhythmic genes and related regulators from the current integrative analysis of multi-omics will facilitate our further understanding on the regulatory mechanism of diurnal rhythms in the liver.

## Materials and methods

### Data source

To determine the rhythmicity of gene expression in different layers, we obtained raw datasets of gene transcription (14) and mature RNA (15) and collected expression of protein (16–18). Moreover, we also gathered the ribosome profiling (Ribo-seq) dataset for qualifying RNA translation rate (19) and proteomic datasets for DBP (18) and protein phosphorylation (20). Only proteins detected in all three independent datasets (14–16) were considered for downstream analysis to get highly confident expressed proteins in the liver. The raw data was extracted from publicly available sources, as indicated in **Table 1**. All datasets were selected from mouse liver tissue that was harvested from multiple time points across light-dark cycles.

**Table 1:** Omics data sets across TTFL.

Profile	Library	Time point	Reference
Transcription	GRO-seq	8	(14)
Mature RNA	RNA-seq	8	(15)
Protein	MS	8, 8, 8	(16–18)
DBP	CatTFRE pull-down +MS	8	(18)
Enhancer RNA	GRO-seq	8	(14)
Translation rate	RNA-seq; Ribo-seq	8;12	(15, 19)
Phosphorylation	MS	8	(20)

GRO-seq, Global Run-On Sequencing. MS, Mass spectrometry. DBP, DNA binding protein. CatTFRE, concatenated tandem array of the consensus TFREs. Ribo-seq, Ribosome profiling.

### Global run-on sequencing data processing

Raw reads were trimmed using fastp v0.23.1 (21). Clean reads were then mapped to the mouse genome (mm10) using Bowtie2 v2.4.1 (22). Samtools v1.14 (23) was used to extract unique mapped reads, followed by the generation of bigwig files using Homer v4.9 (24), which were further visualized on Integrative Genomics Viewer (IGV) (25).

### RNA-seq data processing

RNA-seq data was processed following the pipeline described previously (9, 26, 27). Briefly, raw reads were trimmed using fastp v0.23.1 (21). Clean reads were then mapped to the mouse genome (mm10) using Hisat2 v2.1.0 (28) with default parameters. Unique reads were extracted using samtools v1.14 (23). Read counts were calculated and normalized to reads per kilobase of exon per million reads mapped (RPKM) using Homer v4.9 (24).

### Ribo-seq data processing

Ribo-seq data was processed following the pipeline described previously (19). Briefly, adapter sequences were removed using cutadapt v4.0 (29) with parameters, -a ;AGATCGGA-AGAGCACACGTCTGAACTCCAGTCAC –match-read-wildcards -m 6, followed by further trimming and size-filtered (lengths 26-35 nt) using fastp v0.23.1 (21). Trimmed reads were mapped to the mouse genome (mm10) using Bowtie2 v2.4.1 (22). Unique reads were extracted using samtools v1.14 (23). Read counts were calculated and normalized to reads per kilobase of exon per million reads mapped (RPKM) using Homer v4.9 (24). The translation rate was calculated using an in-house Perl script which has been deposited on GitHub at https://github.com/ChunjieJiang/Multi-Omics_CircadianRhythm_RegulatoryLayers.

### Quantification of transcription rate

We used pre-RNA abundance to quantify the transcription rate. The pre-RNA identified from GRO-Seq was measured following the pipeline described previously (14, 15). Briefly, transcripts were measured using GRO-Seq unique mapped reads. For genes with annotated body length > 12kb, a 10kb window (+2kb to 12kb) was considered for quantification. Genes with annotated body length between 2kb and 12kb were quantified using the window from +2kb to the transcription end site (TES). While for genes shorter than 2kb, the whole gene body was used to do the quantification. Reads from each gene were normalized to reads per kb per ten million reads (RPKTM).

### Quantification of protein from western blot

As a technological limitation of mass spectrometry, some clock genes (e.g., *Bmal1* and *Rev-erb*) could not be detected. The diurnal rhythms of these proteins were indicated by western blot from multiple studies (30–32). Image J (version 1.51, NIH) was used to quantify the relative expression in protein levels across light-dark cycles based on the data from Western Blot.

### *De novo* identification of enhancer RNA

Enhancer RNAs were identified following the pipeline described previously (14, 33). Briefly, unique mapped reads from GRO-Seq were separated into the reads mapped to the plus and minus strands, followed by peak calling using Homer v4.9 (24). Peaks with FDR < 0.001 and fold changes > 3 were considered. Sites located within 300bp of annotated TSSs were excluded. Reads mapped within 500bp away from an enhancer RNA locus center were extracted and normalized to RPKTM.

### Identification of oscillation genes

To determine the rhythmic transcription, mature RNA, protein, DNA binding protein (DBP), enhancer RNA, and translational rate, JTK_CYCLE tests (34) were performed with period range 20-28 h and the amplitude and phase as free parameters. The JTK_CYCLE algorithm calculates the p-value using Kendall’s tau correlation, and then the p-values would be adjusted by Bonferroni correction. This allowed for thorough identification of circadian patterns. Rhythmic transcription, mature RNA, enhancer RNA, and translational rate were defined as those with JTK_CYCLE adj-p value < 0.05. For protein and DNA-binding protein, the statistical cut-off of JTK_CYCLE adj-p value < 0.1 was used as suggested by Wang et al. (18).

## Results

### Strategy to dissect regulatory layers for diurnal gene expression

**Figure 1:**
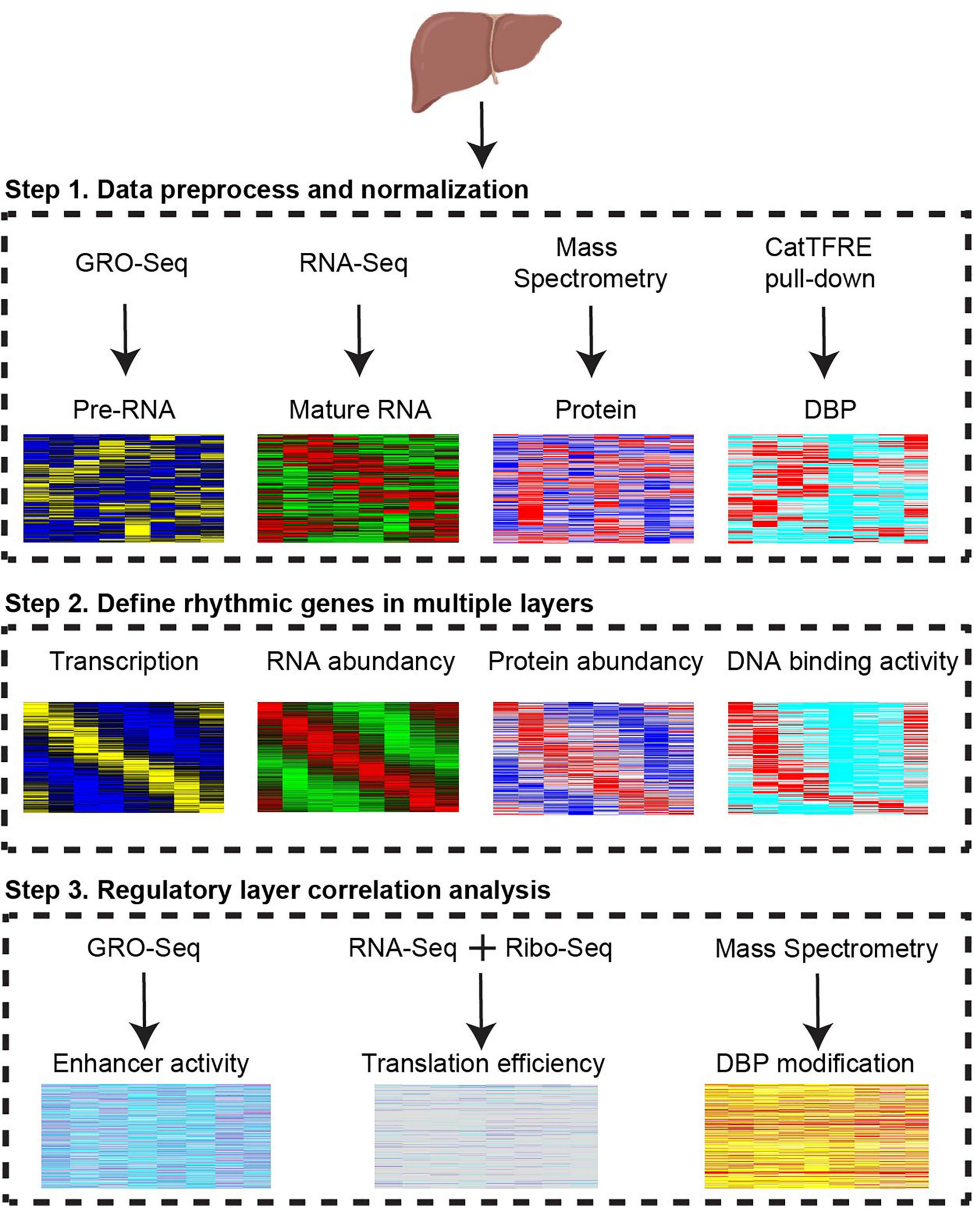
Strategy to dissect regulatory layers for diurnal gene expression. Multi-omics profiles from transcription, mature RNA, protein, DNA binding protein, enhancer RNA, translation rate, and phosphorylation were integrated to reveal multiple rhythmic regulatory layers. CatTFRE, concatenated tandem array of the consensus TFREs. DBP, DNA binding protein. GRO-seq, Global Run-On Sequencing. Ribo-seq, Ribosome profiling.

To systematically investigate the complexity and specificity of diurnal gene expression in different regulatory layers, we first quantified the expression level of RNA transcription, mature RNA abundance, and protein abundance, as well as DNA binding activity of DBP (**Figure 1**). The rhythmicity analysis pipeline was uniformed by using JKT_CYCLE. To explore the potential mechanisms of the specificity of oscillating genes in each layer, we determined the impacts of enhancer activity, translation efficiency, and protein post-translational modification in the counterpart adjacent layers. In sum, this pipeline allows us to globally investigate the full spectrum of gene expression regulatory layers.

### Identification of rhythmic genes in each regulatory layer

**Figure 2:**
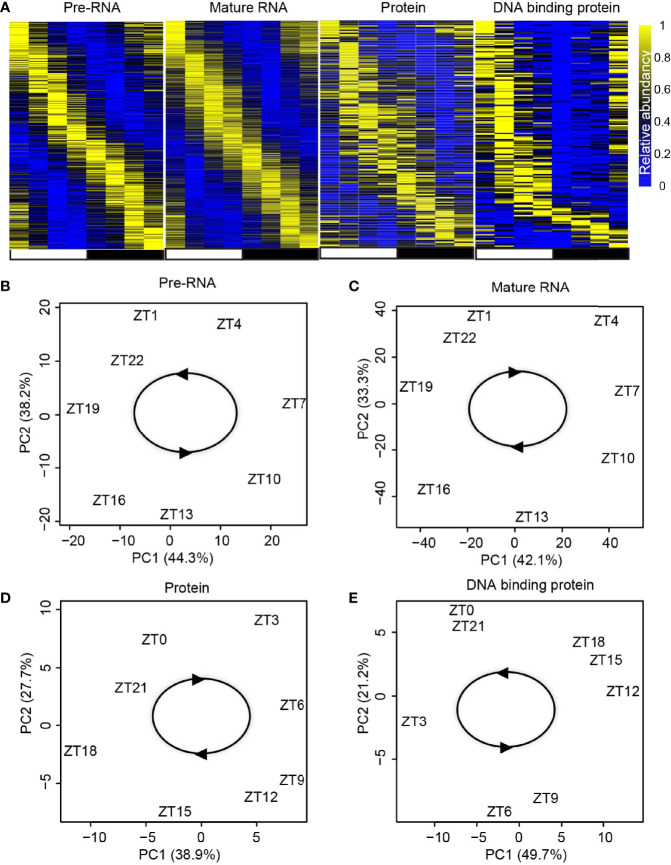
Identification of rhythmic genes in each regulatory layer. **(A)** Heatmap shows the expression/activity of rhythmic genes at transcription, mature RNA, protein, and DBP levels. **(B–E)** Principal component analysis (PCA) for the dataset from transcription **(B)**, mature RNA **(C)**, protein **(D)**, and DBP **(E)** levels.

With the above pipeline, the rhythmic gene transcription, mature RNA abundance, protein abundance, and DPB with rhythmic DNA binding activity were identified (**Figure 2A**). The rhythms of these genes in these four layers were further confirmed using principal component analysis (PCA). The temporally annotated samples in each layer were correctly ordered with non-supervised information (**Figures 2B–E**).

**Figure 3:**
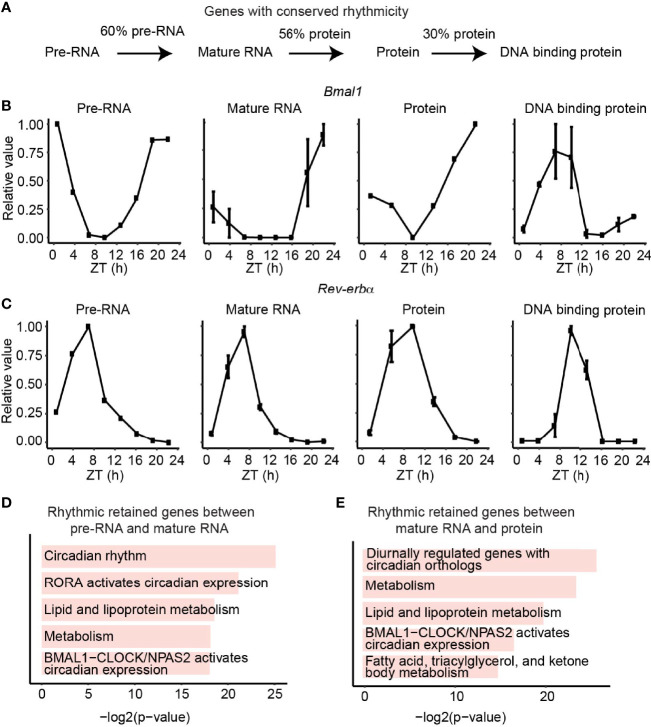
Rhythmic retained genes between adjacent layers. **(A)** The fraction of conserved rhythmic genes between adjacent layers. **(B, C)** The expression/activity of conserved rhythmic gene *Bmal1* **(B)** and *Rev-verbα* (C) at transcription, mature RNA, protein, and DBP level. **(D, E)** Bar plots show the enriched pathways of conserved rhythmic genes between transcription and mature RNA levels **(D)**, and the conserved rhythmic genes between mature RNA and protein levels **(E)**. Only genes detected in both adjacent layers were considered.

Interestingly, we observed 60% of genes with rhythmic transcription stayed rhythmic as mature RNA and 56% of rhythmic protein retained rhythmicity from mature RNA, while only 30% of DBP kept rhythmic DNA binding activity (**Figure 3A**). The core clock genes exampled by *Bmal1* and *Rev-erbα* maintain their rhythmicity across all four layers (**Figures 3B, C**). To further check the function of the reserved rhythmic genes between adjacent layers, functional enrichment analysis was performed using Enrichr (35) based on BioPlanet (36). As expected, pathways involving circadian rhythm and lipid metabolism were enriched for genes with reserved rhythmicity between pre-RNA and mature RNA levels, as well as the ones between mature RNA and protein levels (**Figures 3D, E**).

### Layer-specific rhythmic genes support the layer-specific function

**Figure 4:**
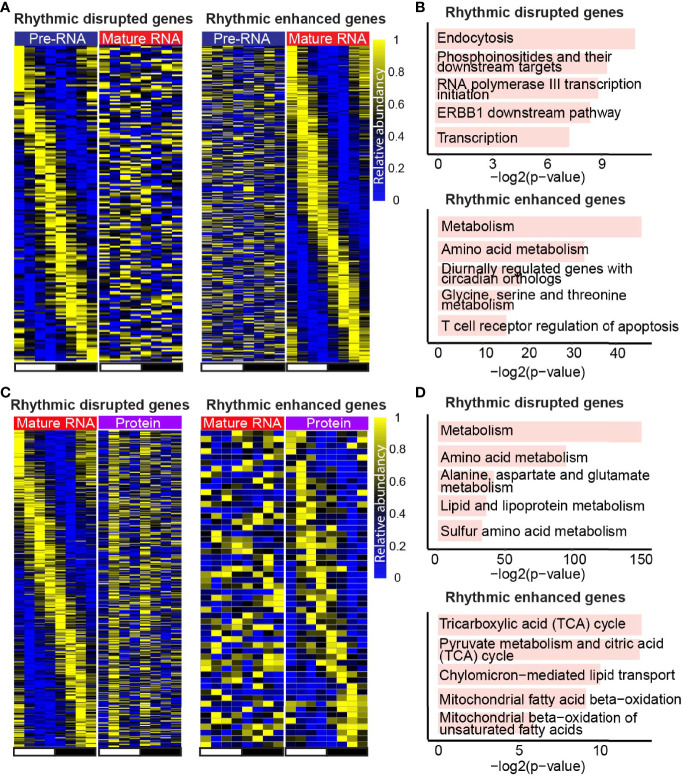
Layer-specific rhythmic genes support the layer-specific function. **(A, C)** Heatmaps show the expression of rhythmic disrupted genes (left panel) and rhythmic enhanced genes (right panel) at mature RNA level compared with transcription **(A)**, and the ones in protein level compared with mature RNA **(C)**. **(B, D)** Bar plots show the enriched pathways of rhythmic disrupted genes (top panel) and rhythmic enhanced genes (bottom panel) in mature RNA level compared with transcription **(B)**, and the ones in protein level compared with mature RNA **(D)**. Only genes detected in both adjacent layers were considered.

Remarkably, besides the conserved rhythmic genes between adjacent layers, we also observed many rhythmic disrupted or enhanced genes in a specific layer. Moreover, genes with rhythmic signals in transcription rate, and non-rhythmic signals in mature RNA level (**Figure 4A**, left panel) were involved in the pathway related to transcription, endocytosis, etc. (**Figure 4B**, upper panel). Conversely, genes with enhanced rhythmic signals in mature RNA level (**Figure 4A**, right panel) were enriched in metabolism and amino acids metabolism biological process (**Figure 4B**, bottom panel). Further analysis on the comparison between mature RNA and protein levels showed that genes playing a function in biological processes such as metabolism and amino acids metabolism (**Figure 4D**, upper panel) were rhythm disrupted genes in protein level (**Figure 4C**, left panel), whereas rhythm enhanced genes (**Figure 4C**, right panel) were involved in TCA cycle and mitochondrial fatty acid metabolism (**Figure 4D**, bottom panel). Altogether, in addition to the rhythmic conserved genes and pathways, there are other regulatory mechanisms mediating the layer-specific oscillating genes.

### Translation rate, post-translational modification, and epigenetic effect contribute to the layer-specific diurnal rhythm

**Figure 5:**
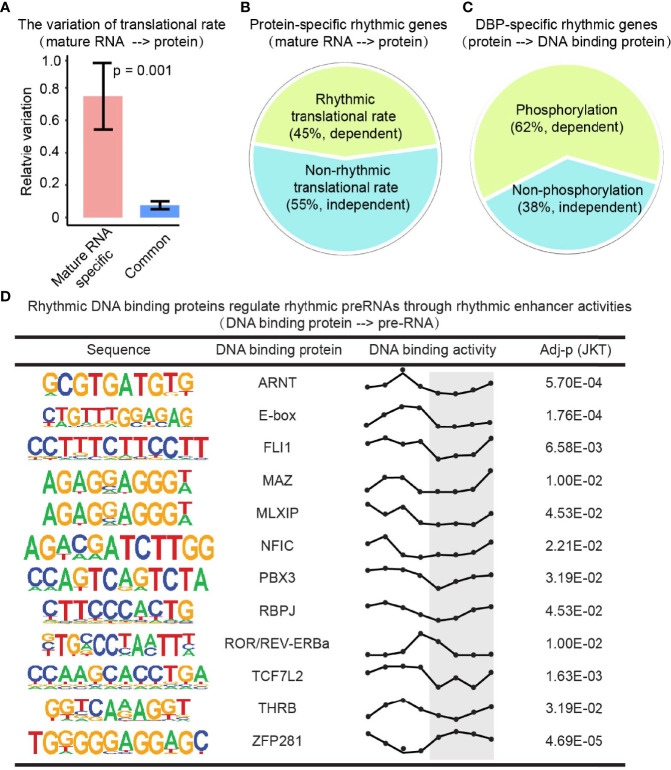
Translation rate, post-translational modification, and epigenetic effect contribute to the layer-specific diurnal rhythm. **(A)** Bar plot shows the variation of translation rates of mature RNA-specific rhythmic genes and the conserved rhythmic genes. P value comes from Student’s t-test. **(B)** Protein-specific rhythmic genes with rhythmic (olive green) or non-rhythmic (cadet blue) translational rate. **(C)** DBP-specific rhythmic genes that were phosphorylated (olive green) or not (cadet blue). **(D)** The DBPs with rhythmic activities regulate rhythmic transcription through rhythmic enhancer activities. The Y axis in the third column present the DNA binding activities. And the X axis present the Zeitgeber time (ZT), a standardized 24-hour notation of the phase in an entrained circadian cycle. ZT0 is the time that light is turned on and ZT12 is the time that light is turned off.

To extend our understanding on the changes of rhythmic expression between mature RNA and protein levels, bulk RNA-seq and Mass Spectrometry data were integrated with Ribo-seq, a technique that can be used to determine translation efficiency (37, 38). The genes with conserved rhythmic expression in both levels showed a much lower variation of translation rate compared to the ones with disrupted rhythmic expression in protein level (**Figure 5A**), implying that genes with rhythmic expression in both mature RNA and protein layers were largely due to the relatively consistent translation rate. Moreover, 45% of the protein-specific rhythmic genes were rhythmic translational rate dependent (**Figure 5B**), indicating that rhythmic translational rate induces the rhythms of protein whose mature RNA levels were not rhythmic.

Protein undergoes post-translational modifications (e.g., phosphorylation) to control its stability, activity, interaction, nuclear localization, and function in different biological processes (39, 40). By integrating the phosphorylation profile, we found that 62% of DBP-specific rhythmic genes are phosphorylation-dependent (**Figure 5C**). Enhancers are known to initiate the transcription of nearby or distal genes together with DBP (e.g., transcriptional factors). To interpret the rhythmic gap from DBP to transcription, enhancer RNA profile from the GRO-seq dataset was investigated. We found there were many rhythmic DBP regulating rhythmic transcription rate through rhythmic enhancer activities (**Figure 5D**). This data indicated a phosphorylation-dependent and an enhancer-dependent regulation on rhythmic expression between the adjacent layers.

## Discussion

Oscillating gene expression is essential for diurnal rhythmic physiology (41, 42). Previous studies on the regulation of oscillating gene expression focused on the regulatory mechanisms between adjacent layers, including transcription rate-mature RNA (19), mature RNA-protein abundance (17), and protein-DNA binding actives pairs (18). The current study provided a full spectrum of regulatory layers on oscillating gene expression, from enhancer activity, transcription, translation, and post-translation modification to the DNA binding activity of DBP. The identification of layer-specific oscillating genes indicates the underlying layer-specific regulatory mechanisms, including RNA-processing, translation efficiency control, post-translational modification, and enhancer actives. Further global studies about RNA stability and protein stability could provide additional insights for layer-specific regulation of oscillating gene expression.

Our integrative analysis of multiple omics represents an example of an in-depth dissection of the complexity and specificity of diurnal rhythms. This comprehensive pipeline has proved to be a powerful tool and enabled us to identify related pathways and potential regulators in each layer. These results are supported by different oscillating omics datasets in mouse livers: (1) epigenomics and transcriptomics profiling identified the regulatory layers between enhancer and gene transcription; (2) transcriptomics and proteomics profiling defined the regulatory layers between mature RNA and protein; (3) ribosome profiling was integrated to determine the potential mechanism for the rhythmic remodeling between above two layers; (4) protein, DBP, and phosphoproteomics profiling comprehensively indicated the role of post-translational modification of DBP activities; 5) DBP proteomics and epigenomics profiling determined the rhythmic regulation of transcription factors on enhancer activities. These omics form a regulatory loop for diurnal rhythmic gene expression. Future hypothesis-driven functional studies are required to determine the layer-specific regulators, which could provide novel targets for the manipulation of diurnal rhythms.

## Data availability statement

The datasets presented in this study can be found in publications. The full citations of these publications can be found in the article/supplementary material.

## Author contributions

CJ and DG conceptualized the study, interpreted data, and wrote the manuscript, which was approved by all authors. PL helped generate part of the omics profiles. CL assisted with writing and technical support. All authors contributed to the article and approved the submitted version.

## Funding

This work was supported by Cancer Prevention and Research Institute of Texas (RR210029, DG), as well as by National Institutes of Health grants (K01-DK125602, DG and pilot award DK056338, DG).

## Conflict of interest

The authors declare that the research was conducted in the absence of any commercial or financial relationships that could be construed as a potential conflict of interest.

## Publisher’s note

All claims expressed in this article are solely those of the authors and do not necessarily represent those of their affiliated organizations, or those of the publisher, the editors and the reviewers. Any product that may be evaluated in this article, or claim that may be made by its manufacturer, is not guaranteed or endorsed by the publisher.
